# Nutrient removal and microalgal biomass production from different anaerobic digestion effluents with *Chlorella* species

**DOI:** 10.1038/s41598-019-42521-2

**Published:** 2019-04-16

**Authors:** Hyeonjung Yu, Jaai Kim, Changsoo Lee

**Affiliations:** School of Urban and Environmental Engineering, Ulsan National Institute of Science and Technology (UNIST), 50 UNIST-gil, Eonyang-eup, Ulju-gun, Ulsan, 44919 Republic of Korea

## Abstract

Potential of microalgal cultivation as an alternative approach to the treatment of anaerobic digestion (AD) effluents was examined using two representative *Chlorella* species, *Chlorella vulgaris* (CV) and *Chlorella protothecoides* (CP). Both species effectively removed NH_4_^+^-N from the AD effluents from four digesters treating different wastes under different operating conditions. In all experimental cultures on the AD effluents, NH_4_^+^-N (initial concentration, 40 mg/L) was completely removed within 10 days without residual NO_3_^−^-N or NO_2_^−^-N in batch mode. Compared to CP, CV showed greater biomass and lipid yields (advantageous for biodiesel production), regardless of the media used. Prolonged nitrogen starvation significantly increased the lipid accumulation in all cultures on the AD effluents, and the effect was more pronounced in the CV than in the CP cultures. On the other hand, compared to CV, CP showed significantly faster settling (advantageous for biomass harvesting) in all media. Our results suggest that the *Chlorella* cultivation on AD effluents under non-sterile, mixed-culture conditions may provide a viable way to manage and valorize the problematic effluents. Diverse bacteria derived from the AD effluents co-existed and presumably interacted with the *Chlorella* species in the cultures.

## Introduction

With growing concerns about energy crisis and global warming, the need for sustainable energy sources is steadily increasing. Converting waste organics into biogas (primarily methane and carbon dioxide) through anaerobic digestion (AD) has been gaining momentum in recent years more as a renewable energy technology than simply as a treatment option^1^. Although AD is widely used in practice, it still has some practical issues to be resolved for more robust applications. Foremost among them is the high ammonia content of the treated effluents from AD processes, which can cause serious eutrophication problems in water bodies if not properly treated before discharge. Conventional biological nutrient removal (BNR) processes, such as A2O process, have been widely used for nitrogen control in wastewater treatment for decades. Although it has been demonstrated as effective, conventional BNR has two major drawbacks that weaken the economic application of the process: the high consumption of oxygen and the need for an external carbon source. These limitations are of even greater concern when treating wastewater with high ammonia nitrogen concentration and low available organic content, such as AD effluents^2^.

Several alternative BNR processes, for example, shortcut nitrification-denitrification, simultaneous nitrification-denitrification, and anammox, have been developed and tested to provide a more feasible treatment option^3–5^. Recently, microalgae-based processes have attracted much attention as a viable alternative for BNR from wastewater. Microalgae can utilize nutrients from wastewater, while photosynthetically fixing carbon dioxide, a greenhouse gas, for growth^6^. This indicates the potential of microalgae-based treatment as an economically as well as environmentally appealing method for BNR, without the need for an external carbon source. Several studies have investigated the application of microalgae to the treatment of AD effluents, and various microalgal species have shown effective removal of nitrogen and phosphorus (>83%) from different AD effluents^7–10^.

Microalgal biomass has been considered a potential feedstock for producing biodiesel, as microalgae accumulate large amounts of assimilated carbon in the form of lipids in the cell. Microalgae have several advantages as a biodiesel feedstock over conventionally used terrestrial plants, including higher lipid content, faster growth, higher biomass yield, and little competition for arable land^11–13^. However, substantial amounts of water and nutrients (often added as chemical salts) are required for the cultivation of microalgae, raising concern over its economic feasibility^14^. Therefore, cultivating microalgae using AD effluents as a medium can provide a more sustainable option for producing energy feedstock, while simultaneously treating nutrient-rich wastewater. The benefits of microalgal cultivation on wastewater can be maximized by performing the cultivation under non-sterile, mixed-culture conditions. A point to consider in such mixed-culture processes is that the nutrient removal and microalgal growth properties will vary not only with the microalgal species (i.e., inoculum) and the characteristics of wastewater (i.e., substrate). Such effects can be more pronounced in cultures using effluents from complex mixed-culture systems such as AD processes. However, the literature addressing this complication is limited. Hence, the present study focuses on this gap and aims to compare different AD effluents for the cultivation of microalgae in terms of nutrient removal and biomass production.

In this study, two representative *Chlorella* species, *Chlorella vulgaris* and *Chlorella protothecoides*, were tested for their ability to remove nutrients (particularly ammonia nitrogen) from and grow on four AD effluents from different sources. *Chlorella* species have been reported to grow well with wastewater nutrients and produce high levels of lipids^6,15^. *C*. *vulgaris* and *C*. *protothecoides* both have promising potential in commercial applications for food, nutrition, and energy purposes, and their ability to grow both autotrophically and heterotrophically could contribute to further degradation of organic residues in AD effluents^16^. Both species have shown greater biomass production and higher lipid productivity or content in the presence of organic carbon sources than in autotrophic cultures^17,18^, which indicates another potential benefit of using AD effluent as a culture medium. For a comprehensive understanding, besides the nutrient removal efficiency, microalgal growth and lipid accumulation were also monitored during cultivation. In addition, the differences in microbial community structure between the *Chlorella* cultures treating different AD effluents were comparatively analyzed by molecular fingerprinting. This is a unique study in that it involves a set of physicochemical and biochemical approaches that allow for a holistic view of microalgal cultivation, from growth monitoring to kinetic modeling, lipid production, and microbial community structure, for complex AD effluents of different characteristics.

## Materials and Methods

### Microalgal strains and pre-cultivation

*Chlorella vulgaris* KCTC AG10002 (CV) and *Chlorella protothecoides* UTEX 1806 (CP) were purchased from the Korean Collection for Type Cultures and the Culture Collection of Algae at the University of Texas at Austin, respectively. CV was pre-cultivated in a modified Bristol medium (BT) with NH_4_^+^-N instead of NO_3_^−^-N as the nitrogen source, whereas CP was cultured in a modified Proteose medium prepared by adding 1 g/L Proteose peptone to BT. BT contained (per liter distilled water (DW)) 157 mg NH_4_Cl, 25 mg CaCl_2_·2H_2_O, 75 mg MgSO_4_·7H_2_O, 75 mg K_2_HPO_4_, 175 mg KH_2_PO_4_, 25 mg NaCl, and 1 mL trace element solution. The trace element solution contained (per liter DW) 0.49 mg Co(NO_3_)_2_·6H_2_O, 8.82 mg ZnSO_4_·7H_2_O, 1.44 mg MnCl_2_·4H_2_O, 0.71 mg MoO_3_, 1.57 mg CuSO_4_·5H_2_O, 11.42 mg H_3_BO_3_, 50 mg EDTA, 31 mg KOH, 4.98 mg FeSO_4_·7H_2_O, and 0.001 mL H_2_SO_4_ (98%, v/v). The prepared media were sterilized by autoclaving at 121 °C for 15 min prior to inoculation. Pre-cultivation was performed in a 500-mL Erlenmeyer flask (working volume, 450 mL) at room temperature (around 20–25 °C) under white light-emitting diode (LED) illumination in a cycle of 16-h light (1000 lux) and 8-h darkness. Each flask was aerated by bottom bubbling with ambient air (170 mL/min) under vigorous magnetic stirring (200 rpm). Cultivation was continued until the NH_4_^+^-N in the culture was exhausted.

### Microalgal cultivation tests with AD effluents

The AD effluents used in this study were collected from four different lab-scale anaerobic digesters treating food waste at a high (HF; 5 g VS/L·d) and a low (LF; 1.5 g VS/L·d) organic loading rates, *Ulva* (UL) and whey (WH), respectively. The operating conditions of the four digesters are summarized in Supplementary Table 1. The effluent samples were taken at a steady state and centrifuged in a 50-mL conical tube at 3400 × *g* for 20 min. The supernatant was recovered and centrifuged again in the same manner. The final supernatant was diluted to a NH_4_^+^-N concentration of 40 mg/L with DW and tested for cultivating the *Chlorella* strains in parallel with BT as the control medium for comparison. The NH_4_^+^-N concentration in the AD effluent media was set to be the same as the nitrogen concentration in BT, one of the most commonly used synthetic medium for the cultivation of *Chlorella*, to investigate the potential use of the AD effluents as a culture medium for microalgal cultivation in comparison with a conventionally used synthetic medium. The nutrient and organic concentrations in the prepared media and the pre-cultivated CV and CP inocula are presented in Supplementary Table 2.

The microalgal cultivation tests with the prepared media were performed under white LED illumination of 1000 lux with a 16-h light/8-h dark cycle in batch mode. Each cultivation trial was run in a 500-mL Erlenmeyer flask with a working volume of 400 mL. The pre-cultivated microalgae were used as seed culture to inoculate the reaction mixtures at a volume/volume ratio of 10%. The flasks were incubated under the same aeration and mixing conditions as the pre-cultivation. All cultivation tests were performed in duplicate at room temperature without pH control and sampled daily to monitor the nutrient removal and microalgal growth.

### Physicochemical analyses

Chemical oxygen demand (COD) was colorimetrically analyzed using HS-COD-MR kit (HUMAS), and solids were measured according to the procedures in Standards Methods^19^. Anions and cations were measured using two ion chromatographs (Dionex ICS-1100, Thermo Scientific) equipped with an IonPac AS14 column and an IonPac CS12A column, respectively. Samples for ion analysis were prepared by filtration through a syringe filter with 0.22 μm pore size.

The microalgal growth was monitored by measuring the optical density at a wavelength of 680 nm (OD_680_) as well as the chlorophyll concentration during the batch cultivation. The total chlorophyll concentration (sum of chlorophyll a and b) was determined as previously described^20^. Briefly, 1 mL of microalgal culture was sampled and centrifuged at 12,000 × *g* for 10 min, and the supernatant was replaced by 1 mL of dimethyl sulfoxide to extract the pigments. The pigment-containing sample was then incubated at 65 °C for 60 min and centrifuged at 12,000 × *g* for 10 min. The concentrations of chlorophyll a and b were estimated from the absorbance at 649 nm (Abs_649_) and 665 nm (Abs_665_) of the resulting supernatant using the following equations.

1 $${\rm{Chlorophyll}}\,{\rm{a}}\,{\rm{concentration}}\,({\rm{mg}}/{\rm{L}})=12.47{{\rm{Abs}}}_{665}-3.62{{\rm{Abs}}}_{649}$$

2 $${\rm{Chlorophyll}}\,{\rm{b}}\,{\rm{concentration}}\,({\rm{mg}}/{\rm{L}})=25.06{{\rm{Abs}}}_{649}-6.5{{\rm{Abs}}}_{665}$$

The total lipid concentration was measured by colorimetric sulfo-phospho-vanillin (SPV) method as previously described^21^, and the lipid content of biomass (%, w/w) was calculated based on the concentration of volatile suspended solids. Fatty acid composition was determined by the direct fatty acid methyl ester (FAME) synthesis method. FAMEs were prepared from dried microalgal biomass by direct transesterification of fatty acids as previously described^22^, and analyzed using a gas chromatograph (7820A, Agilent) coupled with a flame ionization detector and an Innowax column (Agilent). A standard FAME mix (C_4_–C_24_, Supelco) was used to identify and quantify FAMEs based on their retention times. All physicochemical analyses were carried out at least in duplicate.

### Microalgal growth and nutrient consumption modeling

The observed chlorophyll accumulation and NH_4_^+^-N removal profiles were modeled using the following modified Gompertz equations:

3 $${{\rm{C}}}_{{\rm{t}}}={{\rm{C}}}_{{\rm{P}}}\cdot \exp [-\exp \{\frac{{{\rm{P}}}_{{\rm{m}}}\cdot {\rm{e}}}{{{\rm{C}}}_{{\rm{P}}}}(\lambda -{\rm{t}})+1\}]$$

4 $${{\rm{N}}}_{0}-{{\rm{N}}}_{{\rm{t}}}={{\rm{N}}}_{0}\cdot \exp [-\exp \{\frac{{{\rm{R}}}_{{\rm{m}}}\cdot {\rm{e}}}{{{\rm{N}}}_{0}}(\lambda -{\rm{t}})+1\}]$$

where C_t_ is the total chlorophyll concentration (mg/L) at time t, C_P_ is the maximum potential of chlorophyll production (mg/L), P_m_ is the maximum chlorophyll production rate (mg/L·d), N_0_ is the initial NH_4_^+^-N concentration (mg/L), N_t_ is the residual NH_4_^+^-N concentration at time t (mg/L), R_m_ is the maximum NH_4_^+^-N removal rate (mg/L·d), λ is the lag phase length (d), and t is the cultivation time (d).

### Biomass harvesting efficiency

The harvesting efficiency of the cultivated biomass by gravity settling was determined as described previously by Darpito *et al*.^7^. An 8-mL volume was taken from each culture and vigorously shaken in an 11-mL glass vial (diameter, 1 cm; height, 14 cm). The optical density at a wavelength of 540 nm (OD_540_) of each culture sample was measured directly from the vial 2 cm above the bottom. The cultures were measured at 5, 15, 30 and 60 min for careful monitoring of settling profiles during the early period and at longer, irregular intervals thereafter along the profiles (3, 6, 10 and 14 h for the CV cultures, and 2, 4, 6, 7, 11, 25 h for the CP cultures). For each measured point, the harvesting efficiency was calculated using the following equation:

5 $${\rm{Harvesting}}\,\mathrm{efficiency}\,( \% )=\frac{O{D}_{0}-O{D}_{t}}{O{D}_{0}}\times 100$$

where OD_0_ is the sample OD_540_ at time zero, and OD_t_ is the sample OD_540_ at time t.

### Prediction of fuel properties

The average degree of unsaturation (ADU) of microalgal oil was estimated based on the fatty acid composition according to Hu *et al*.^23^, using the following equation:

6 $${\rm{ADU}}={\rm{\Sigma }}M\times {{\rm{Y}}}_{{\rm{i}}}$$

where M is the number of carbon-carbon double bonds in each fatty acid constituent, and Y_i_ is the mass fraction of each fatty acid constituent. The fuel properties of biodiesel transformed from the extracted microalgal oil were predicted as described previously, using the equations listed below^23^.

7 $${\rm{Cetane}}\,{\rm{number}}\,({\rm{CN}})=-\,6.6684\times {\rm{ADU}}+62.876$$

8 $${\rm{Cloud}}\,{\rm{point}}\,({\rm{CP}})=-\,\,13.356\times {\rm{ADU}}+19.994$$

9 $${\rm{Higher}}\,{\rm{heating}}\,{\rm{value}}\,({\rm{HHV}})=1.7601\times {\rm{ADU}}+38.534$$

10 $${\rm{Iodine}}\,{\rm{value}}\,({\rm{IV}})=74.373\times {\rm{ADU}}+12.71$$

11 $${\rm{Kinematic}}\,{\rm{viscosity}}\,({\rm{KV}})=-\,0.6316\times {\rm{ADU}}+5.2065$$

12 $${\rm{Specific}}\,{\rm{gravity}}\,({\rm{SG}})=0.0055\times {\rm{ADU}}+0.8726$$

### Molecular fingerprinting

Total DNA was extracted from the microalgal culture samples using an automated nucleic acid extractor (ExiProgen, Bioneer) as previously described^24^. Denaturing gradient gel electrophoresis (DGGE) analysis was performed with the prepared DNA samples to look at the microbial community structures. Bacterial 16S rRNA genes and eukaryotic 18S rRNA genes were amplified by polymerase chain reaction (PCR) using BAC338F/805 R and EUK1A/516 R primer sets, respectively, using a touch-down thermal protocol^24^. The resulting PCR amplicons were electrophoresed on 8% (v/v) polyacrylamide gels with 20–60% (bacteria) or 10–50% (eukaryotes) denaturant gradients for 16 h at 80 V in a D-code system (Bio-Rad, USA). The gels were stained with SYBR Safe dye (Molecular Probe) after electrophoresis and visualized under blue light transillumination. Bands of interest were retrieved from the gels for further sequencing analysis as previously described^24^. The obtained gene sequences were compared against the GenBank database using the BLAST program for phylogenetic affiliation. All nucleotide sequences reported in this study have been deposited in the GenBank database under accession numbers MH155232-MH155241.

### Statistical analysis of DGGE fingerprints

The bacterial and eukaryotic DGGE fingerprints were each converted into a matrix based on the relative contribution of individual bands to the total band intensity in each lane, using TotalLab 1D image processing software (TotalLab). The constructed matrices were analyzed by cluster analysis using the unweighted pair group method with arithmetic means (UPGMA) to visualize the relatedness between the analyzed microbial community structures. Clustering calculations and dendrogram generation, with the Bray-Curtis distance measure, were carried out using PAST 3.17^25^.

## Results and Discussion

### Nutrient removal

**Figure 1:**
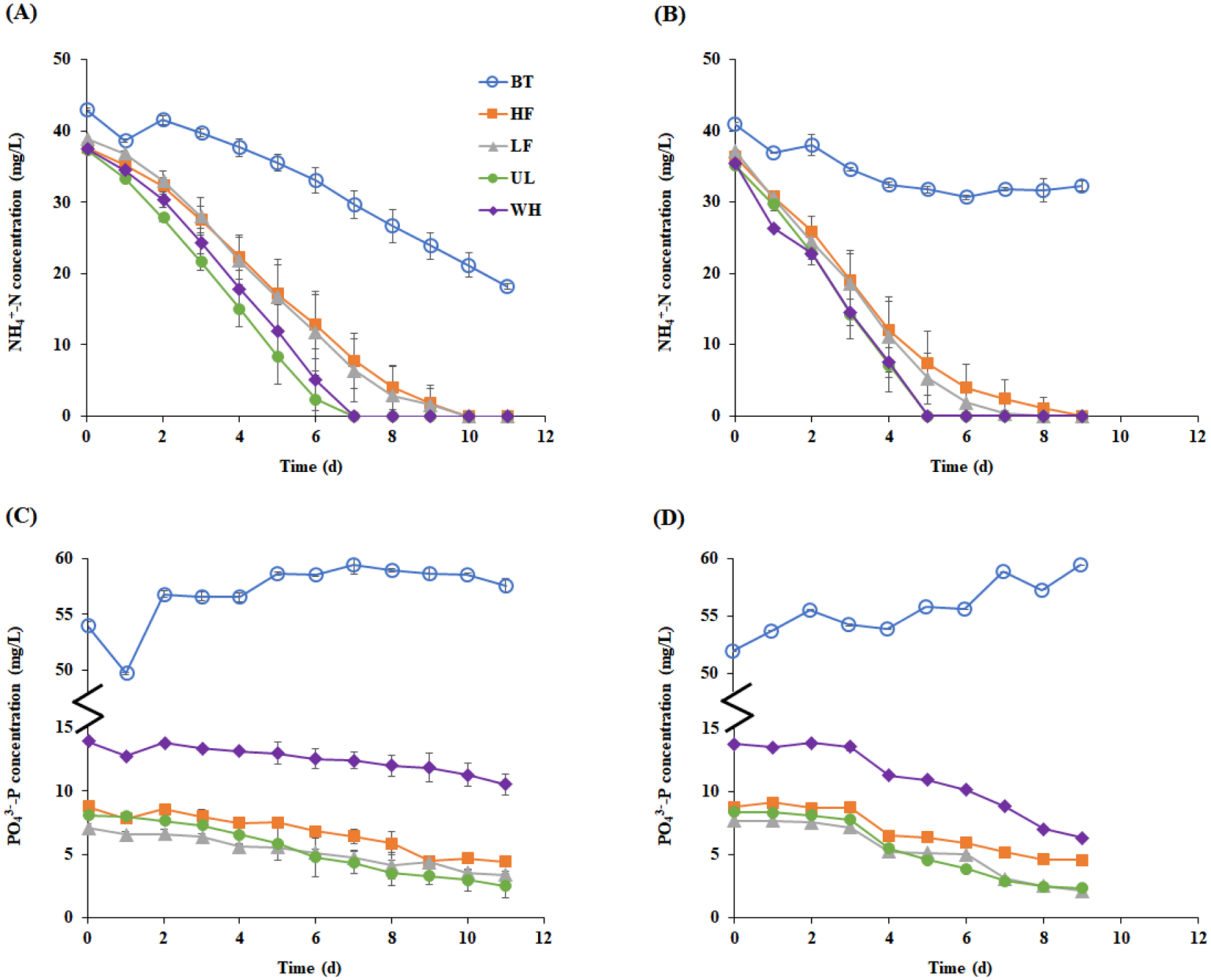
Residual concentrations of NH_4_^+^-N and PO_4_^3−^-P in *Chlorella vulgaris* (**A** and **C**) and *Chlorella protothecoides* (**B** and **D**) cultures. The curves are labeled with the corresponding culture media: one synthetic (Bristol medium, BT) and four prepared using effluents from different anaerobic digesters treating food waste at a high (HF; 5 g VS/L·d) and a low (LF; 1.5 g VS/L·d) organic loading rates, *Ulva* (UL), and whey (WH), respectively. Results are expressed as mean ± standard deviation (n = 2).

NH_4_^+^-N was completely removed within 10 days of cultivation in all the cultures on AD effluents, while its residual concentration remained at high levels (>20 mg/L) in the cultures on BT, for both CV and CP (Fig. 1A,B). This can be related to the low N/P ratio of BT (<1) (see Supplementary Table 2). N/P ratio is an important factor that affects not only nutrient removal but also the extent of microalgal growth. The optimal N/P ratio may vary with substrate characteristics or microalgal strains, and it has been reported that *Chlorella* species grow well with efficient removal of nutrients at an N/P ratio between 5 and 15^26–28^. In this study, the N/P ratios of the AD effluents were in or near this range, which presumably contributed to the effective removal of NH_4_^+^-N.

For all the AD effluents tested, CP achieved the complete removal of NH_4_^+^-N in less time than CV, whereas NH_4_^+^-N in BT was consumed faster by CV than by CP. Correspondingly, the maximum NH_4_^+^-N removal rate (R_m_) was significantly higher (10–22%) in the CP cultures than in the CV cultures for all AD effluents (Table 1). Interestingly, for both *Chlorella* species, the culture on UL showed the highest R_m_ (8.5 mg/L·d for CV and 10.4 mg/L·d for CP), followed by those on WH, LF, and HF. On the other hand, the maximum specific NH_4_^+^-N removal rate (q_m_), calculated by dividing R_m_ by the initial chlorophyll concentration, was higher in the CV cultures than in the CP cultures. For instance, when cultivated on UL, R_m_ was 8.5 mg/L·d for CV and 10.4 mg/L·d for CP, whereas q_m_ was 9.0 d^−1^ for CV and 6.3 d^−1^ for CP. These results indicate that, although chlorophyll is a rough measure of microalgal biomass, CV might have performed superior to CP in terms of the specific activity (per cell or unit biomass) to remove NH_4_^+^-N from the AD effluents. The observed q_m_ values (4.1–11.5 d^−1^ for the CV cultures and 4.7–7.2 d^−1^ for the CP cultures) are significantly higher than previously reported values for *Chlorella* species cultivated on synthetic or real wastewaters (≤3.8 d^−1^, calculated based on chlorophyll a only)^29,30^. For both species, the lag phase was significantly longer when cultivated on BT (≥3.1 days) than on the AD effluents (≤1.7 days) (Table 1). CP showed shorter lag periods (<1 day) than CV did for all AD effluents, which could be due to the higher initial microalgal concentration in the CP cultures, as mentioned above. It is notable that CP achieved only marginal NH_4_^+^-N removal, with a much longer lag time compared with CV, when cultivated on BT. This appears to be attributable to the absence of organic carbon sources in BT (see Supplementary Table 2). CP is metabolically versatile, can grow both autotrophically and heterotrophically, and is reported to grow significantly better under heterotrophic conditions^31^. This explains why CP showed much more effective and faster NH_4_^+^-N removal in the AD effluents than in BT. Another possibility is that CP may be more sensitive than CV to a low N/P ratio of the substrate.

**Table 1:** Kinetic parameters of nitrogen removal and chlorophyll production estimated using modified Gompertz equations in the *Chlorella* cultures.

Parameter	Unit	CV					CP				
		BT	HF	LF	UL	WH	BT	HF	LF	UL	WH
Maximum nitrogen removal rate (R_m_)	mg/L·d	3.1	6.3	6.9	8.5	8.4	nd^a^	7.5	8.4	10.4	9.2
Lag phase (λ)	d	3.1	1.7	1.6	1.2	1.6	nd	0.6	0.6	0.9	0.6
Maximum specific nitrogen removal rate (q_m_)	d^−1^	4.1	5.0	6.9	9.0	11.5	nd	4.7	5.9	6.3	7.2
Chlorophyll production potential (C_P_)	mg/L	23.9	25.5	25.9	13.8	17.0	5.4	12.0	16.8	13.3	14.3
Maximum chlorophyll production rate (P_m_)	mg/L·d	2.0	2.9	3.1	2.6	3.5	1.1	2.0	2.2	1.6	2.2
Lag phase (λ)	d	1.0	1.2	1.1	1.5	1.8	—^b^	—	—	—	—

*Note*: *Chlorella vulgaris* (CV) and *Chlorella protothecoides* (CP) were cultivated on five media: one synthetic (Bristol medium, BT) and four prepared using effluents from different anaerobic digesters treating food waste at a high (HF; 5 g VS/L·d) and a low (LF; 1.5 g VS/L·d) organic loading rates, *Ulva* (UL), and whey (WH), respectively.

^a^Not determined due to insignificant nitrogen removal.

^b^Not observed.

In all *Chlorella* cultures on the AD effluents, PO_4_^3−^-P was removed alongside NH_4_^+^-N during the cultivation (Fig. 1C,D). PO_4_^3−^-P removal was also much more rapid and significant in the cultures with AD effluents, and there was almost no removal of PO_4_^3−^-P in the cultures on BT, for both CV and CP. The PO_4_^3−^-P removal efficiency was comparable or higher in CP (48–72%) than in CV (24–69%). The nutrient removal profiles showed that both CV and CP were effective in the microalgal treatment of residual nutrients, particularly NH_4_^+^-N, in the AD effluents.

### Microalgal growth

**Figure 2:**
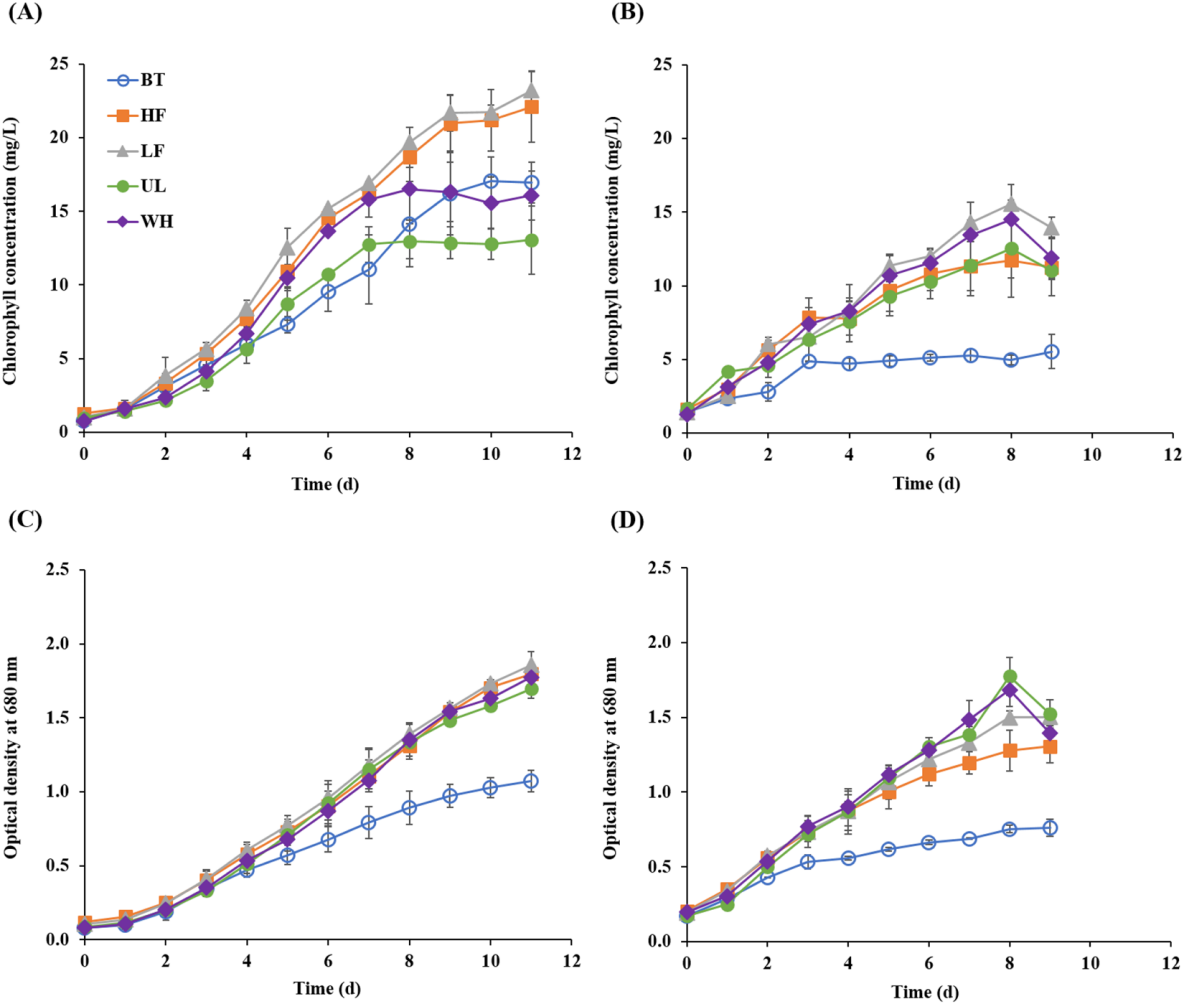
Biomass growth measured as chlorophyll concentration and optical density in *Chlorella vulgaris* (**A** and **C**) and *Chlorella protothecoides* (**B** and **D**) cultures. The curves are labeled with the corresponding culture media: one synthetic (Bristol medium, BT) and four prepared using effluents from different anaerobic digesters treating food waste at a high (HF; 5 g VS/L·d) and a low (LF; 1.5 g VS/L·d) organic loading rates, *Ulva* (UL), and whey (WH), respectively. Results are expressed as mean ± standard deviation (n = 2).

Both NO_3_^−^ and NO_2_^−^ concentrations remained negligible (<1 mg/L) during the cultivation under aerobic conditions (data not shown), suggesting that the removed NH_4_^+^-N was mostly assimilated into biomass, given the aerobic culture conditions. Figure 2 shows the temporal changes in total chlorophyll concentration and OD_680_, which are widely used to estimate microalgal abundance, for the microalgal cultures. Although both the chlorophyll and OD_680_ levels increased with the consumption of NH_4_^+^-N with time, the two indicators showed significantly different temporal profiles, particularly during the later period of cultivation. This appears to be because OD_680_ cannot differentiate between microalgal cells and other microorganisms or even non-biological suspended particles, potentially leading to confounding results. Such errors can be more pronounced in non-sterile, mixed-culture systems, which are prone to bacterial contamination. Therefore, microalgal growth was monitored using the concentration change in chlorophyll to avoid such errors in this study, although OD_680_ may better represent the total biomass.

Both CV and CP grew well without a substantial lag on the AD effluents (Fig. 2A,B). This indicates that the microalgae readily adapted to the AD effluents and utilized the nutrients, resulting in the short lag periods in NH_4_^+^-N removal (Table 1). Proper dilution of AD effluents is an important factor to consider when using them for microalgal cultivation. If the medium is too dense, the light penetration is limited, whereas if it is too dilute, the nutrient levels are insufficient^32^. In this study, the effluents were diluted to a NH_4_^+^-N concentration of 40 mg/L, which appears to have provided favorable conditions for the growth of both *Chlorella* species.

Interestingly, for both CV and CP, the chlorophyll accumulation patterns did not correlate well with the NH_4_^+^-N removal patterns. CV showed markedly greater cumulative production of chlorophyll in the cultures on LF and HF, but the NH_4_^+^-N consumption was the slowest than in other cultures (Figs 1A and 2A). The amount of chlorophyll produced in the CV cultures on UL and WH was even lower compared with the culture on BT, which showed incomplete removal of NH_4_^+^-N in the medium. These results suggest that CV grew with different yields (per NH_4_^+^-N removed) in the cultures on different media. This is supported by the modeling results, where different CV cultures showed comparable maximum chlorophyll production rates (P_m_), but significantly different values for maximum potential of chlorophyll production (C_P_) (Table 1). Such differences in apparent yield might be associated in part with the potential of NH_4_^+^-N consumption by bacteria co-existing with the microalgal species in the experimental cultures. Although less pronounced, similar trends were observed in the CP cultures. The culture on LF showed the highest production of chlorophyll, followed by those on WH, UL, and HF, which is very different from the order of their NH_4_^+^-N removal rates (UL > WH > LF > HF). Unlike in the CV cultures, the chlorophyll production in the CP culture on BT was much lower than those in the cultures on the AD effluents, correlating well with the NH_4_^+^-N removal results (Figs 1B and 2B). This can be related to the characteristics of CP, which, as mentioned above, grows better in heterotrophic or mixotrophic cultures^31^.

The increase in chlorophyll concentration stopped upon the exhaustion of NH_4_^+^-N, and no apparent growth was observed afterwards, in most of the cultures on the AD effluents, except the CP cultures on UL and WH. The growth of CP after NH_4_^+^-N exhaustion can be explained by the ability of microalgae to uncouple nutrient uptake from growth with internal nutrient storage^33,34^. On the other hand, OD_680_ continued to increase for a while after NH_4_^+^-N exhaustion in all cultures on the AD effluents (not seen clearly in the CP culture on HF within the cultivation period). This contrast can be attributed to the growth of bacteria, particularly of heterotrophic bacteria (using cell debris and extracellular secretion as carbon and nitrogen sources) or nitrogen-fixing bacteria, which can cause significant errors in the measurement of microalgal biomass based on optical density.

The culture experiments demonstrated that both CV and CP grew well on all the AD effluents tested and, in most cases, showed significantly higher rates of microalgal growth and NH_4_^+^-N removal. This could be partially attributed to the presence of residual organic compounds in AD effluents, which could promote the growth of microalgae^35,36^. Our results suggest a great potential of microalgal cultivation on AD effluents, not only for the treatment of AD effluents but also for the production of microalgal biomass.

### Biomass settling

Harvesting cultivated biomass accounts for at least 20–30% of the total costs in producing microalgal biomass, and therefore, the biomass settleability is an important factor to consider, particularly in large-scale cultivation^7,37^. *Chlorella* species reportedly have good auto-settling properties, which, although not completely clarified, seems to be influenced by ecophysiological factors such as pH, ions, and co-existing bacterial species^38,39^. The microalgal culture samples for auto-settling tests were taken on days 11 and 9 for the CV and CP cultures, respectively. CV and CP showed completely different settling behaviors (see Supplementary Fig. 1). The CV cultures on the AD effluents had lag periods of several hours and significantly slower settling rates than those on BT. However, all CV cultures reached comparable high harvesting efficiencies (83.5–93.1%) in 24 h. On the other hand, the CP cultures settled quickly without lag, thus showing significantly greater initial settling rates than those of the CV cultures. The CP cultures showed high harvesting efficiencies of 76.6–89.6% in 25 h, with more than 85% of the maximum harvesting efficiency determined at 25 h being achieved within 6 h in all CP cultures. These results suggest that CP can have advantage over CV when the time for harvesting (or volume of settling basin) is limited, although all cultures on the AD effluents eventually achieved fairly comparable harvesting efficiencies. Interestingly, for both CV and CP, the highest harvesting efficiency among the cultures on the AD effluents was observed when cultivated on UL. *Ulva* biomass has a high content of metals such as Ca, Fe, and Mg, whose multivalent cations can help to neutralize the negative surface charge of microalgae and promote the aggregation of microalgal cells and other particles^40,41^.

### Lipid production and composition

**Figure 3:**
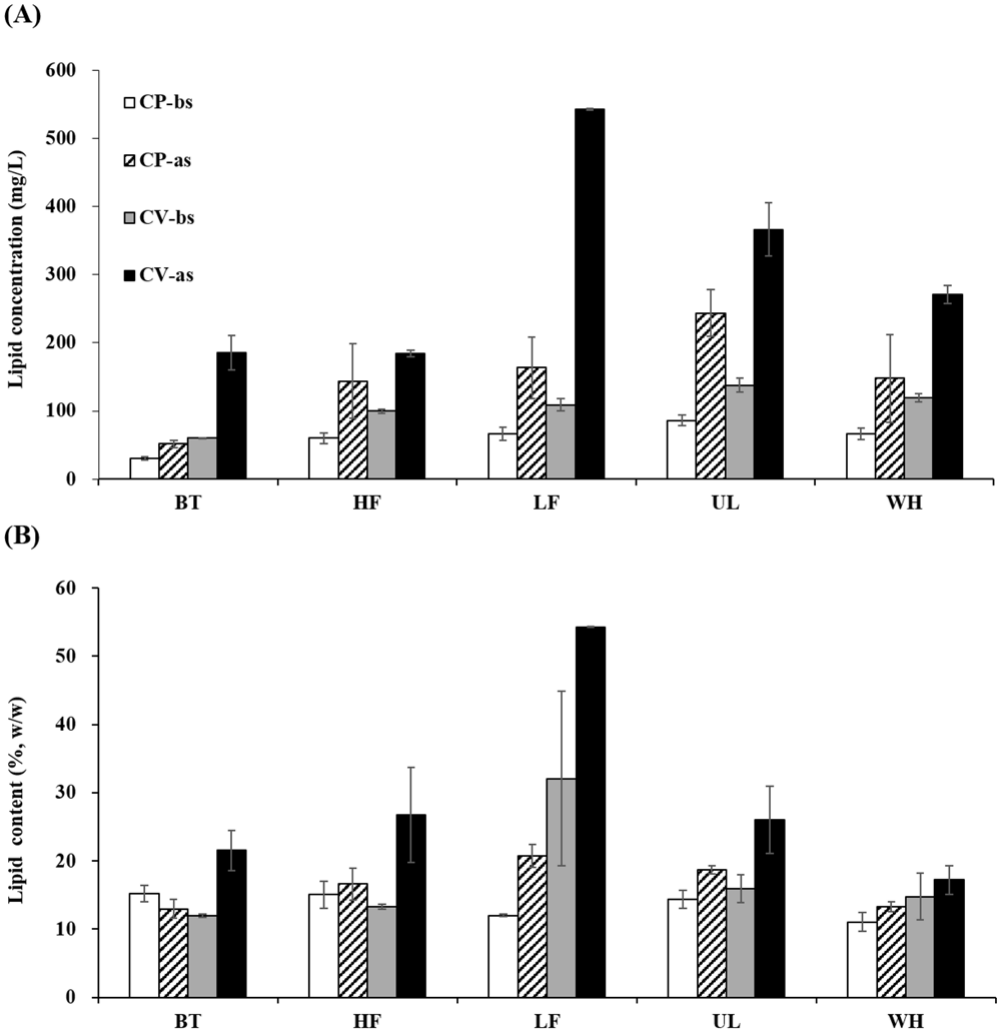
Total lipid concentration (per unit volume of culture; (**A**) and content (per unit mass of biomass; (**B**) in *Chlorella vulgaris* (CV) and *Chlorella protothecoides* (CP) cultures before (-bs) and after (-as) prolonged cultivation under nitrogen starvation. The x-axis represents five different media used for microalgal cultivation: one synthetic (Bristol medium, BT) and four prepared using effluents from different anaerobic digesters treating food waste at a high (HF; 5 g VS/L·d) and a low (LF; 1.5 g VS/L·d) organic loading rates, *Ulva* (UL), and whey (WH), respectively. Results are expressed as mean ± standard deviation (n = 2).

To evaluate the potential for oil recovery, each culture was examined for the lipid content and composition of cultivated biomass. The productivity and properties of lipids vary greatly with microalgal species and culture conditions^42^. Several studies have recently reported that lipid productivity increases under nitrogen starvation^43–45^. Therefore, in this study, the lipids were analyzed for each culture before (i.e., after the complete NH_4_^+^-N removal) and after a nitrogen starvation period of 7 days (i.e., incubation with no additional medium or nutrients): on days 11 and 18 for the CV cultures and on days 9 and 16 for the CP cultures. All cultures on the AD effluents produced greater amounts of lipids compared to those on BT (1.7–2.3-fold difference for CV and 2.0–2.8-fold difference for CP) (Fig. 3A). This may be due to the effect of residual organic carbon in the AD effluents^46^. Both CV and CP showed the largest lipid production on UL before nitrogen starvation (137.8 mg/L for CV and 86.1 mg/L for CP). Interestingly, the lipid concentration increased significantly after the starvation in all cultures. The increase was more pronounced in the CV cultures (1.8–5.0-fold) than in the CP cultures (1.7–2.8-fold), and the largest lipid concentration was observed in the CV culture on LF after nitrogen starvation. These results correspond to previous observations, and a recent study even reported an increase by up to 20-fold in lipid accumulation for *Chlorella sorokiniana* under nitrogen-depleted conditions^43^.

With the increase in lipid accumulation, the lipid content increased in all cultures, except the CP culture on BT after starvation (Fig. 3B). The lipid content ranged from 11% to 15%, which is comparable to those previously reported for *Chlorella*^47^, before starvation in the experimental cultures. After the starvation, it increased by up to 2-fold, with the highest lipid content of 54% observed in the CV culture on LF. This is a significantly greater value than the normal range for autotrophic *Chlorella* cultures (14–30%)^47^. A previous study, although using a synthetic medium, reported a remarkably high lipid content of 89% (of dry weight) for a CV strain under nitrogen-depleted conditions^44^. These suggest that nitrogen starvation can provide a simple way to improve the lipid yield, and thus, the economic feasibility of microalgal biodiesel, particularly when wastewater is used as a culture medium. However, there are several reports of limitation of microalgal growth under nitrogen-depleted condition, which can eventually reduce the total productivity of microalgal lipids^48^. Therefore, careful attention is required to avoid such a negative effect while applying the nitrogen starvation strategy, for example, by employing a two-stage process with separate microalgal growth and lipid production stages^39^.

**Figure 4:**
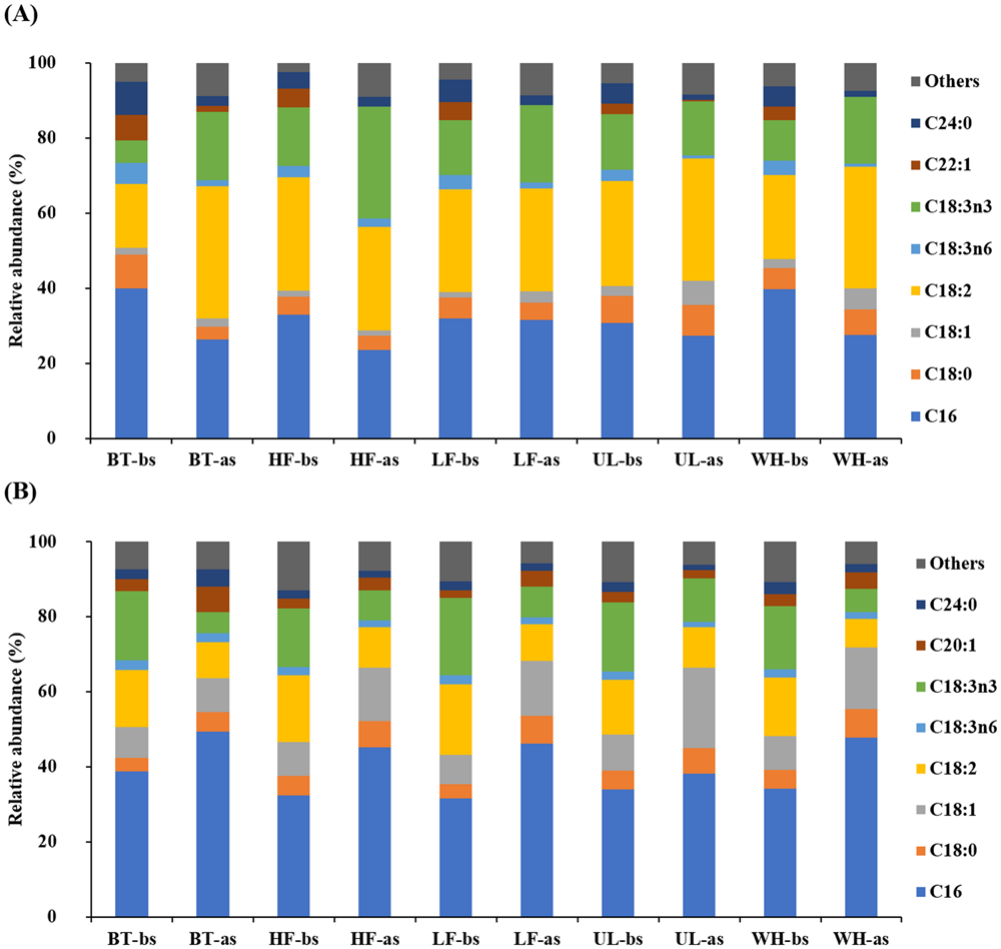
Fatty acid composition of total lipids in *Chlorella vulgaris* (**A**) and *Chlorella protothecoides* (**B**) cultures before (-bs) and after (-as) prolonged cultivation under nitrogen starvation. The x-axis represents five different media used for microalgal cultivation: one synthetic (Bristol medium, BT) and four prepared using effluents from different anaerobic digesters treating food waste at a high (HF; 5 g VS/L·d) and a low (LF; 1.5 g VS/L·d) organic loading rates, *Ulva* (UL), and whey (WH), respectively. Results are expressed as mean ± standard deviation (n = 2).

CV and CP showed significantly different lipid composition, largely due to the difference in the composition of C18 fatty acids (Fig. 4). C16, C18:2, and C18:3n3 were the major fatty acids commonly found in the CV cultures. On the other hand, in the CP cultures, C18:1 formed one of the major C18 fatty acids, in addition to C18:2 and C18:3n3, with C16 being the dominant fatty acid in all cultures. It is notable that the proportion of C16 decreased with an increase in the proportion of C18 fatty acids after nitrogen starvation in the CV cultures, whereas starvation led to a marked increase in the proportion of C16, with a decrease in the proportion of C18 fatty acids in the CP cultures. These results mean that CV and CP had different lipid production characteristics and responded differently to nitrogen depletion stress in the experimental cultures, supporting the fact that the properties of microalgal lipids vary among different strains^42^. Both CV and CP yielded microalgal lipids suitable for biodiesel production, in terms of fatty acid composition, from the AD effluents. Taking into account the content of monounsaturated fatty acids, particularly oleic acid (C18:1), and the ratio of saturated to unsaturated fatty acids (see Supplementary Table 3), lipids from the CP cultures have superior properties for the oxidative stability and low-temperature fluidity of biodiesel^49,50^. Several fuel properties of biodiesel, related to combustion quality and stability, engine performance, low-temperature properties, and heating value, were calculated based on the fatty acid composition (see Supplementary Table 4). All lipid samples from the experimental cultures met the European (EN14214) and American (ASTM D6751) biodiesel standards, further confirming that the CV and CP cultures on the AD effluents produced microalgal lipids suitable for biodiesel production.

### Microbial community structure

**Figure 5:**
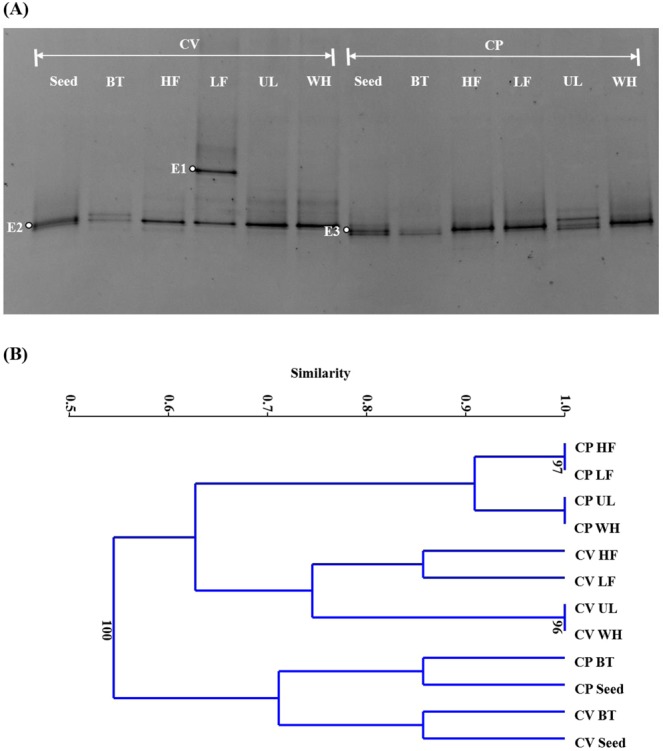
Denaturing gradient gel electrophoresis profiles (**A**) and cluster dendrogram (**B**) of the eukaryotic communities in *Chlorella vulgaris* (CV) and *Chlorella protothecoides* (CP) cultures. The lanes and nodes are labeled with the corresponding microalgal species and culture media: one synthetic (Bristol medium, BT) and four prepared using effluents from different anaerobic digesters treating food waste at a high (HF; 5 g VS/L·d) and a low (LF; 1.5 g VS/L·d) organic loading rates, *Ulva* (UL), and whey (WH), respectively. The *Chlorella* seed cultures are labeled as Seed. The full-length gel for (**A**) is a presented in Supplementary Fig. 2.

The eukaryotic and bacterial communities in the microalgal cultures were characterized by DGGE of total DNA samples taken from each culture before the period of prolonged nitrogen starvation: on day 11 for the CV cultures and on day 9 for the CP cultures (Fig. 1). It is worth noting that several eukaryotic bands other than those in the seed cultures were detected in the cultures on the AD effluents (Fig. 5A). These bands probably represent the eukaryotes derived from the AD effluents, given the non-sterile, mixed-culture conditions of cultivation. A unique eukaryotic band (E1) detected with high intensity in the CV culture on LF was closely related (≥97% sequence similarity) to several *Colpodea* ciliates (Table 2). This suggests that the culture contained high levels of bacteria to support the growth of the bacterivorous protozoan associated with E1^51^. The *Chlorella*-related bands derived from the seed cultures (E2 and E3) appeared as a major band in their corresponding cultures. Both bands showed high similarities (≥90%) to several *Chlorella* species including CV and CP. Although not robustly quantitative, the DGGE fingerprints further support the fact that both the *Chlorella* species adapted to and grew well in the AD effluents.

**Table 2:** Phylogenetic affiliation of eukaryotic 18S and bacterial 16S rRNA gene sequences retrieved from denaturing gradient gel electrophoresis bands.

Domain	Band	Closest relative	Accession No.	Similarity (%)	Query coverage (%)
Eukaryotes	E1	*Colpoda aspera*	KF111344	100	96
		*Kreyellidae* sp. MD-2012	JQ723971	100	96
		*Kalometopia duplicata*	KJ873050	98	96
	E2	*Chlorella* sp. WT1	KX109776	90	100
		*Chlorella vulgaris*	KJ561358	90	100
		*Chlorella saccharophila*	X63505	90	100
	E3	*Chlorella emersonii*	KF673368	99	97
		*Chlorella zofingiensis*	KM985403	97	100
		*Chlorella protothecoides* var. *acidicola*	AJ439399	95	100
Bacteria	B1	*Sphingopyxis macrogoltabida*	NR_113720	99	100
		*Sphingopyxis soli*	NR_116739	99	100
	B2	*Nubsella zeaxanthinifaciens*	NR_114146	99	100
		*Pedobacter nanyangensis*	NR_137392	99	100
	B3	*Blastomonas natatoria*	NR_040824	100	100
		*Sphingomonas ursincola*	NR_040825	100	100
	B4	*Sphingomonas kyeonggiensis*	NR_134182	100	100
		*Sphingomonas leidyi*	NR_025324	100	100
	B5	*Tabrizicola aquatica*	NR_117979	99	100
		*Gemmobacter lanyuensIS*	NR_109450	99	100
	B6	*Carbophilus carboxidus*	NR_104931	99	100
		*Aminobacter aminovorans*	NR_025301	99	100
		*Mesorhizobium cantuariense*	NR_137373	99	100
	B7	*Corynebacterium vitaeruminis*	NR_121721	99	100
		*Corynebacterium ulcerans*	NR_117211	99	100

The eukaryotic cluster dendrogram shows a clear separation of the CV and CP cultures on the AD effluents, implying that seed biomass had more decisive effect than the characteristics of the effluents on the evolution of eukaryotic community during the cultivation (Fig. 5B). On the other hand, the cultures on BT were most closely related to their seed cultures and formed a distinct cluster. This suggests that the type of media (i.e., synthetic or effluent) also had a critical influence on the eukaryotic community structure. A possible reason for such difference is the unintended introduction of eukaryotic microorganisms from the AD effluents.

**Figure 6:**
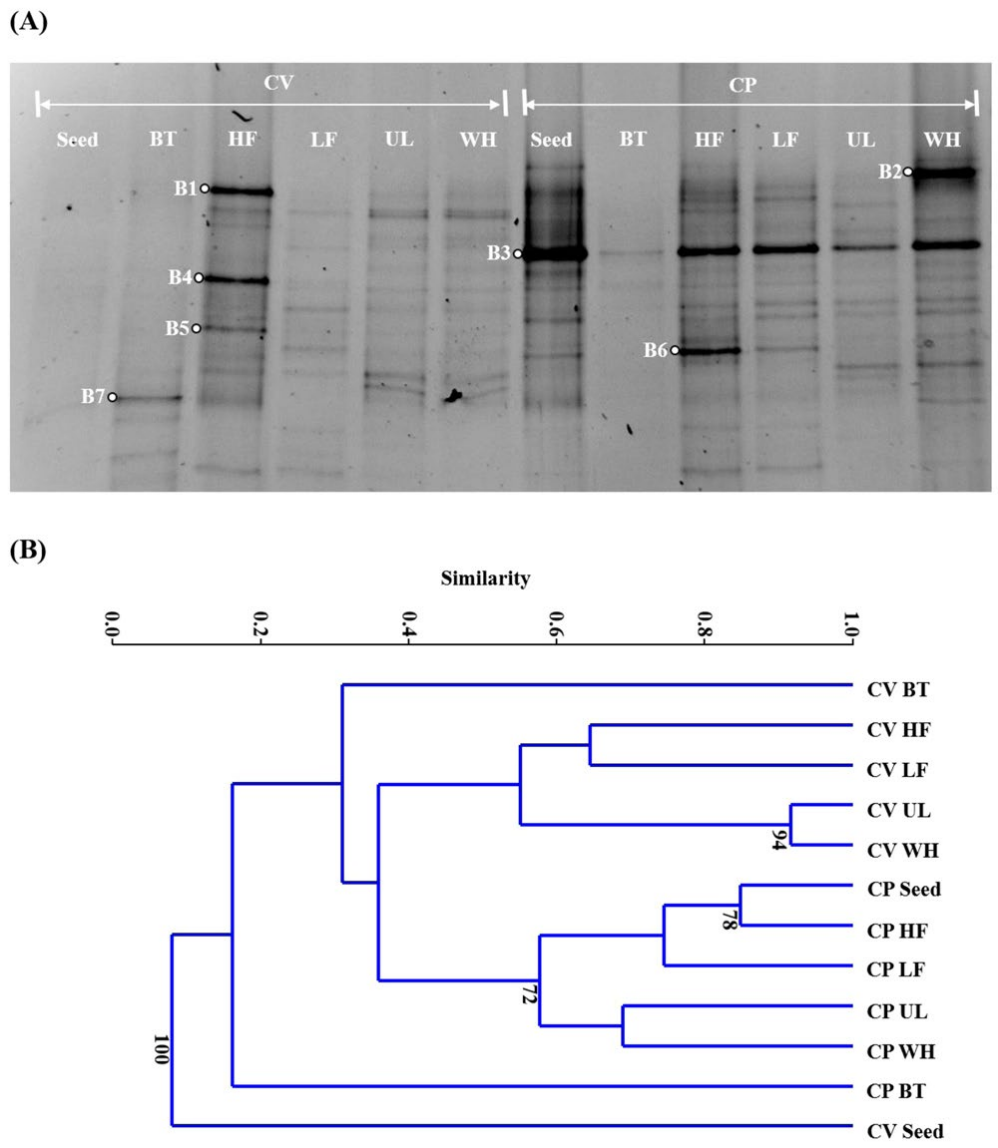
Denaturing gradient gel electrophoresis profiles (**A**) and cluster dendrogram (**B**) of the bacterial communities in *Chlorella vulgaris* (CV) and *Chlorella protothecoides* (CP) cultures. The lanes and nodes are labeled with the corresponding microalgal species and culture media: one synthetic (Bristol medium, BT) and four prepared using effluents from different anaerobic digesters treating food waste at a high (HF; 5 g VS/L·d) and a low (LF; 1.5 g VS/L·d) organic loading rates, *Ulva* (UL), and whey (WH), respectively. The *Chlorella* seed cultures are labeled as Seed. The full-length gel for (**A**) is a presented in Supplementary Fig. 3.

The bacterial DGGE fingerprints showed much more complicated and dynamic structures than the eukaryotic DGGE results (Fig. 6A). The retrieved band sequences (B1 to B7) were all closely related to known bacterial species (Table 2). As in the eukaryotic cluster analysis, the CV and CP cultures on the AD effluents were clearly separated from each other in the bacterial cluster dendrogram (Fig. 6B). This indicates that seed microalgae had the dominant influence on the differentiation of the bacterial community structure. It has already been reported that microalgae interact with many other organisms in their growing environment and affect the diversity of bacterial community^52^. It should be noted that the CP seed culture was contaminated with bacteria, particularly with the bacterium corresponding to B3 (Fig. 6A). B3 was closely related to metabolically versatile *Sphingomonadaceae* species^53^, including *Blastomonas natatoria*, which is a photosynthetic bacterium capable of using peptone as a carbon source^54^. This suggests the possibility that this bacterial species was enriched during the pre-cultivation of CP in a medium containing proteose peptone (refer to Subsection 2.1). This is supported by the fact that B3 appeared as a major band with strong intensity in all CP cultures on the AD effluents, but as a faint band in the culture on BT without organic carbon. This may explain why the CP seed culture was clustered together with the CP cultures on the AD effluents in the cluster analysis (Fig. 6B).

The remaining sequences were assigned to bacterial groups commonly found in various natural and engineered systems: *Sphingomonadaceae* (B1 and B4), *Sphingobacteriaceae* (B2), *Rhodobacteraceae* (B5), *Rhizobiales* (B6), and *Corynebacteriaceae* (B7). B2, detected as the dominant band in the culture on WH (effluent from an anaerobic digester treating lactose-rich whey), was closely related to *Nubsella zeaxanthinifaciens*, which is capable of utilizing lactose^55^. B5 was closely related to the photosynthetic *Rhodobacteraceae* species. Some members of the family are able to accumulate fatty acids^56^, and the bacterium related to B5 may have, in part, contributed to the production of lipids. B6 was closely related to several nitrogen-fixing *Rhizobiales* species, which may have provided nitrogen for other microbes to use in the cultures, particularly under nitrogen starvation conditions^57,58^. The molecular analysis results confirm that microalgae and bacteria co-existed and interacted in the experimental culture. de-Bashan *et al*.^59^ have suggested that the co-immobilized microalgal and bacteria can be beneficial for the growth of microalgae, and other studies also have reported that co-existing bacteria play an important role in nutrient removal in microalgae-based wastewater treatment processes^60,61^. Supply of phytohormones, exchange of carbon and nutrients, and elimination of pathogenic microorganisms have been suggested as possible interactions between microalgae and bacteria that are beneficial for the growth of microalgae^58^.

## Conclusions

CV and CP were tested for their potential to grow on and treat different AD effluents, i.e., HF, LF, UL, and WH, in comparison to a synthetic medium BT, in batch mode. Both CV and CP cultures showed significantly higher nutrient removal efficiencies in the AD effluents than in BT, with the substrate NH_4_^+^-N being completely removed within 10 days of cultivation on all the AD effluents. Correspondingly, both microalgal species generally had better growth on the AD effluents than on BT. Cultivation under nitrogen starvation significantly increased the lipid accumulation in all cultures, regardless of the media used. The increase in lipid content was even more pronounced in the CV than in the CP cultures. The CV cultures demonstrated higher microalgal growth and lipid production, whereas the CP cultures showed better auto-settling properties for biomass harvesting. For all cultures, the fuel properties estimated based on the fatty acid composition proved that the produced lipids were suitable as a feedstock for biodiesel production. The microbial community analysis results showed that both CV and CP adapted and grew well on the AD effluents under non-sterile, mixed-culture conditions in the presence of diverse bacteria. The overall results suggest a high potential for microalgae-based treatment and valorization of AD effluents. A point to consider, particularly in large-scale applications, is the great need for water to dilute AD effluents, which limits the economic feasibility of the microalgal cultivation process. Therefore, further studies on the microalgal adaptation to higher nitrogen loading (or less dilution) conditions and the minimization of water use by harvest water recycling are needed.

## Supplementary information

supplementary information

## Data Availability

All data generated or analyzed during this study are included in this published article and its supplementary information files.
