# Supplementary material for: Nutrient removal and microalgal biomass production from different anaerobic digestion effluents with Chlorella species
Source: Sci Rep. 2019 Apr 16;9:6123. doi: 10.1038/s41598-019-42521-2 (PMC6467878; doi:10.1038/s41598-019-42521-2)
Supplement: Supplementary file 1 — supplementary information [file 41598_2019_42521_MOESM1_ESM.pdf]

Nutrient removal and microalgal biomass production from different anaerobic digestion effluents with *Chlorella* species

Hyeonjung Yu, Jaai Kim, Changsoo Lee*

School of Urban and Environmental Engineering, Ulsan National Institute of Science and Technology (UNIST), 50 UNIST-gil, Eonyang-eup, Ulju-gun, Ulsan 44919, Republic of Korea

* Corresponding author.

Tel.: +82 52 217 2822; fax: +82 52 217 2819

E-mail address: cslee@unist.ac.kr

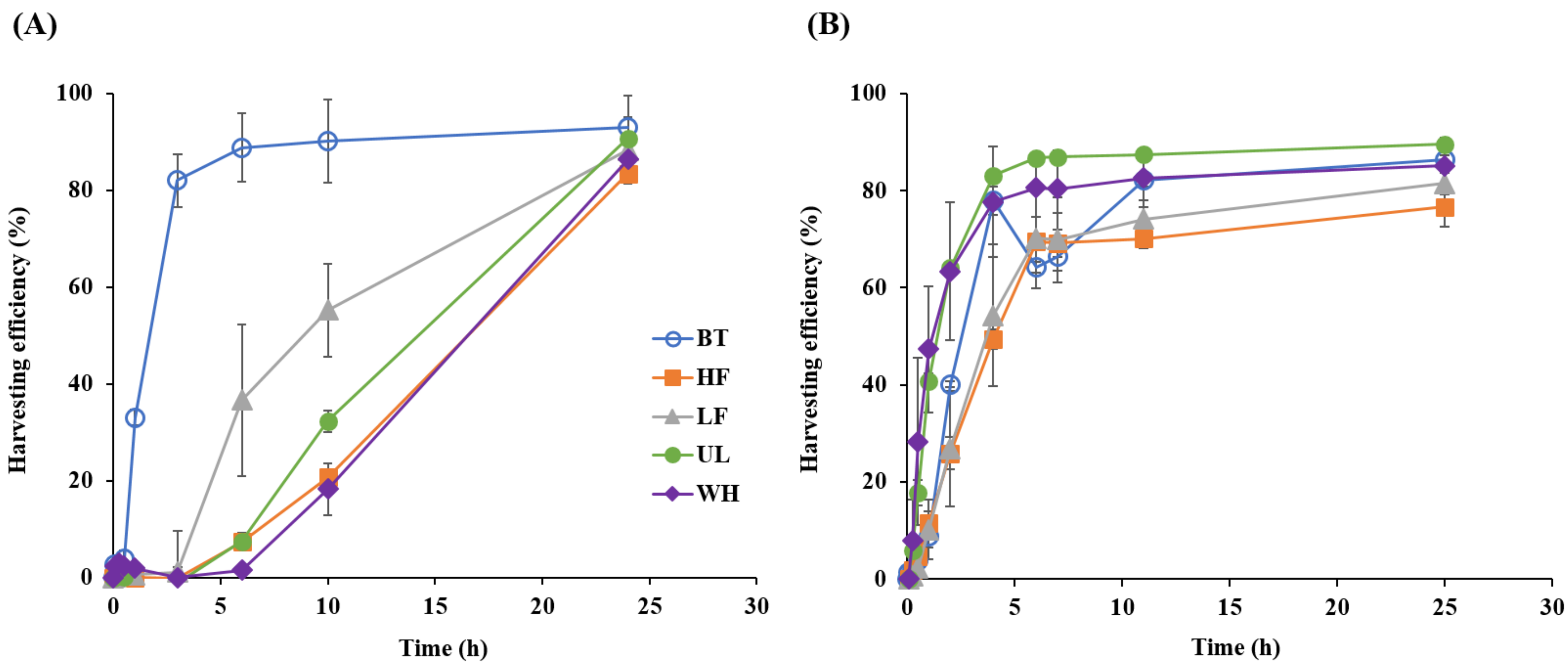

Supplementary Figure 1. Biomass harvesting efficiency by gravity settling for *Chlorella vulgaris* (A) and *Chlorella protothecoides* (B) cultures on five media: one synthetic (Bristol medium, BT) and four prepared using effluents from different anaerobic digesters treating food waste at a high (HF; 5 g VS/L·d) and a low (LF; 1.5 g VS/L·d) organic loading rates, *Ulva* (UL), and whey (WH), respectively. Results are expressed as mean ± standard deviation (n = 2).

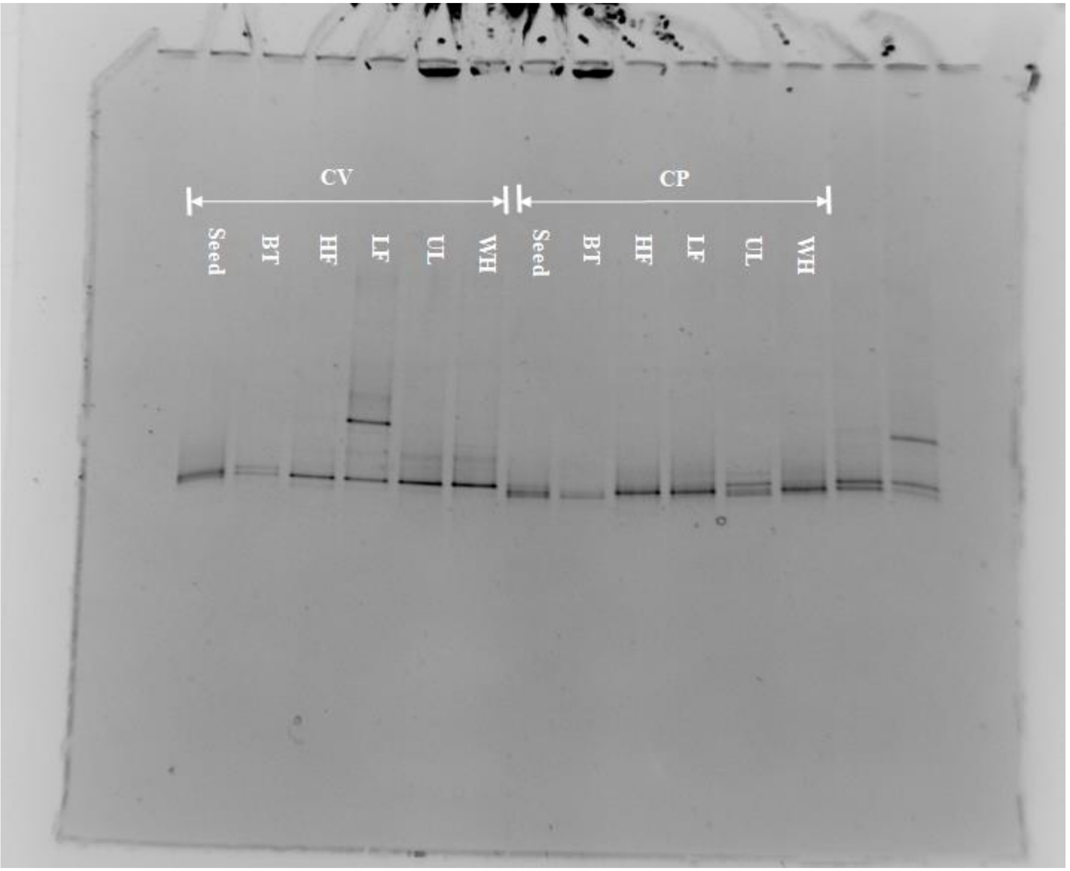

Supplementary Figure 2. Full-length DGGE gel image of the eukaryotic communities in *Chlorella vulgaris* (CV) and *Chlorella protothecoides* (CP) cultures (Fig. 5A). The lanes are labeled with the corresponding microalgal species and culture media: one synthetic (Bristol medium, BT) and four prepared using effluents from different anaerobic digesters treating food waste at a high (HF; 5 g VS/L·d) and a low (LF; 1.5 g VS/L·d) organic loading rates, *Ulva* (UL), and whey (WH), respectively. The *Chlorella* seed cultures are labeled as Seed. The last two lanes were loaded with samples from a different study.

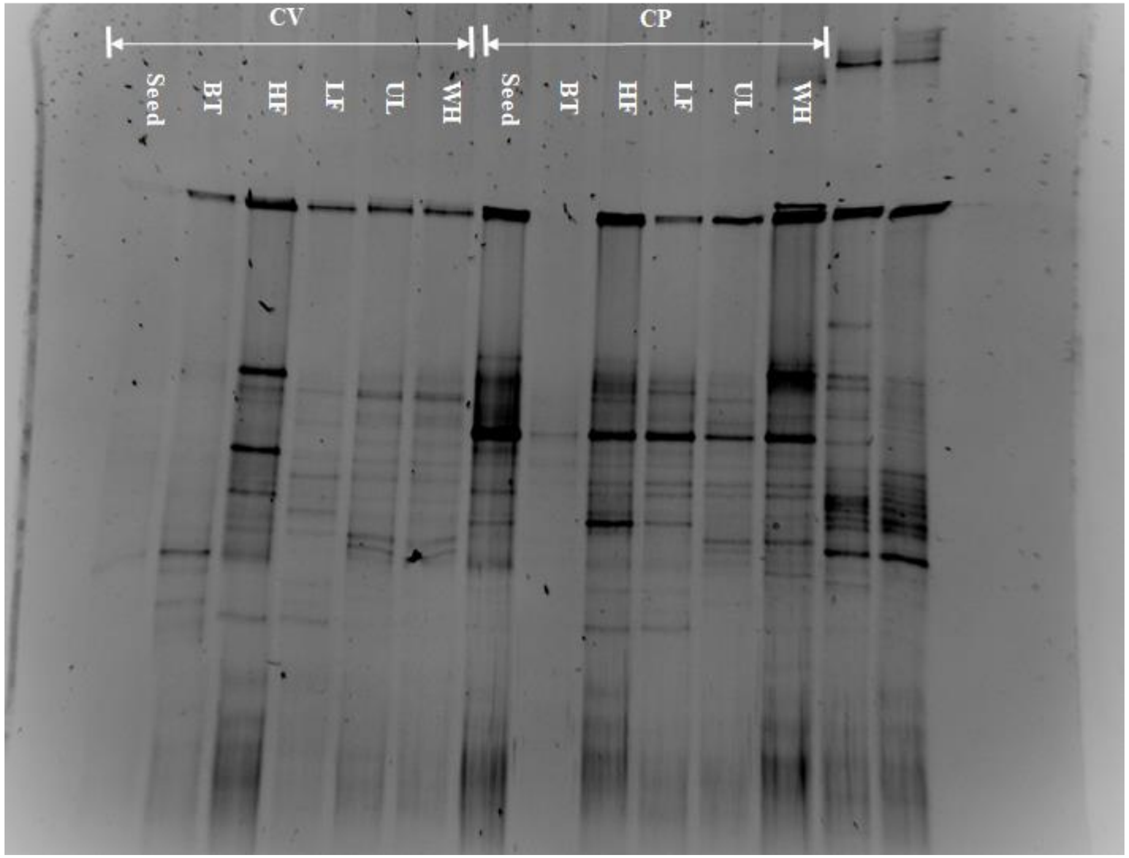

Supplementary Figure 3. Full-length DGGE gel image of the bacterial communities in *Chlorella vulgaris* (CV) and *Chlorella protothecoides* (CP) cultures (Fig. 6A). The lanes are labeled with the corresponding microalgal species and culture media: one synthetic (Bristol medium, BT) and four prepared using effluents from different anaerobic digesters treating food waste at a high (HF; 5 g VS/L·d) and a low (LF; 1.5 g VS/L·d) organic loading rates, *Ulva* (UL), and whey (WH), respectively. The *Chlorella* seed cultures are labeled as Seed. The last two lanes were loaded with samples from a different study.

Supplementary Table 1. Operating conditions and effluent characteristics of four source anaerobic digesters.

Digester	Operating conditions						Effluent characteristics		
	Substrate	Reactor type	HRT (day)	OLR (g/L·d)	Temperature (°C)	Methane yield (L/g)	NH_4^+-N (mg/L)	PO_4^{3-}-P (mg/L)	COD (mg/L)
HF	Food waste	CSTR	20	5.0[a]	35	0.5[a]	1761.0 ± 76.9	147.7 ± 31.0	3636.0 ± 34.0
LF	Food waste	CSTR	60	1.5[a]	35	0.4[a]	2446.3 ± 154.7	98.9 ± 26.7	3069.2 ± 19.0
UL	*Ulva*	CSTR	20	0.3[b]	35	0.2[b]	92.6 ± 1.0	6.3 ± 0.0	279.7 ± 5.0
WH	Whey	CSTR	20	0.3[b]	35	0.3[b]	97.7 ± 1.4	22.8 ± 0.6	68.3 ± 13.0

[a] On a VS basis.

[b] On a COD basis.

Supplementary Table 2. Nutrient and organic concentrations in the culture media and seed cultures.

	NH_4^+-N (mg/L)	PO_4^{3-}-P (mg/L)	COD (mg/L)
BT	46.5 ± 0.0	56.6 ± 0.0	–[a]
HF	42.7 ± 3.6	4.9 ± 1.6	83.4 ± 5.6
LF	41.6 ± 1.3	3.1 ± 1.4	50.9 ± 4.3
UL	38.4 ± 0.5	3.7 ± 1.4	122.3 ± 3.7
WH	38.9 ± 0.1	10.0 ± 1.2	30.2 ± 1.4
CV seed culture	0.00 ± 0.0	46.7 ± 0.1	nd[b]
CP seed culture	0.00 ± 0.0	47.6 ± 0.0	nd[b]

Note: *Chlorella vulgaris* (CV) and *Chlorella protothecoides* (CP) were cultivated on five media: one synthetic (Bristol medium, BT) and four prepared using effluents from different anaerobic digesters treating food waste at a high (HF; 5 g VS/L·d) and a low (LF; 1.5 g VS/L·d) organic loading rates, *Ulva* (UL), and whey (WH), respectively.

[a] Not detected.

[b] Not determined.

Supplementary Table 3. Saturated and unsaturated fatty acid composition (%, w/w) of lipids from the *Chlorella* cultures on different media before (-bs) and after (-as) prolonged cultivation under nitrogen starvation.

Chlorella species	Culture	SFA[a]	MUFA	PUFA	UFA	SFA/UFA ratio
CV	BT-bs	62.8	8.7	28.6	37.2	1.7
	BT-as	35.5	9.5	55.0	64.5	0.6
	HF-bs	42.3	8.8	48.8	57.7	0.7
	HF-as	33.4	5.2	61.4	66.6	0.5
	LF-bs	45.4	8.8	45.8	54.6	0.8
	LF-as	42.0	8.3	49.7	58.0	0.7
	UL-bs	43.3	10.8	45.9	56.7	0.8
	UL-as	40.6	10.0	49.4	59.4	0.7
	WH-bs	54.0	8.9	37.1	46.0	1.2
	WH-as	39.6	9.4	51.0	60.4	0.7
CP	BT-bs	46.4	14.2	39.4	53.6	0.9
	BT-as	61.0	17.3	21.7	39.0	1.6
	HF-bs	41.3	20.5	38.2	58.7	0.7
	HF-as	55.0	22.3	22.7	45.0	1.2
	LF-bs	39.0	16.6	44.4	61.0	0.6
	LF-as	56.6	21.4	22.0	43.4	1.3
	UL-bs	42.2	19.4	38.3	57.8	0.7
	UL-as	47.1	27.5	25.4	52.9	0.9
	WH-bs	43.1	19.0	37.9	56.9	0.8
	WH-as	57.9	24.2	17.9	42.1	1.4

Note: *Chlorella vulgaris* (CV) and *Chlorella protothecoides* (CP) were cultivated on five media: one synthetic (Bristol medium, BT) and four prepared using effluents from different anaerobic digesters treating food waste at a high (HF; 5 g VS/L·d) and a low (LF; 1.5 g VS/L·d) organic loading rates, *Ulva* (UL), and whey (WH), respectively.

[a] SFA, saturated fatty acid; MUFA, monounsaturated fatty acid; PUFA, polyunsaturated fatty acid; UFA, unsaturated fatty acid

Supplementary Table 4. Estimated fuel properties of biodiesel transformed from the lipids from the *Chlorella* cultures on different media before (-bs) and after (-as) prolonged cultivation under nitrogen starvation.

Biodiesel standard or *Chlorella* species	Culture	CN[a]	IV (g I_2/100g)	CP (°C)	KV (mm^2/s)	HHV (MJ/kg)	SG (kg/L)
EN14214		>51	<120	na	3.5-5.0	na	0.86-0.9
ASTM D6751		>47	na[b]	na	1.9-6.0	na	na
CV	BT-bs	57.7	70.3	9.7	4.7	39.9	0.88
	BT-as	53.6	116.4	1.4	4.3	41.0	0.88
	HF-bs	54.5	105.8	3.3	4.4	40.7	0.88
	HF-as	52.5	128.9	-0.9	4.2	41.3	0.88
	LF-bs	55.0	101.2	4.1	4.5	40.6	0.88
	LF-as	54.2	109.5	2.6	4.4	40.8	0.88
	UL-bs	54.8	102.3	3.9	4.5	40.7	0.88
	UL-as	54.8	102.7	3.8	4.4	40.7	0.88
	WH-bs	56.4	85.4	6.9	4.6	40.3	0.88
	WH-as	54.2	109.4	2.6	4.4	40.8	0.88
CP	BT-bs	62.9	12.7	20.0	5.2	38.5	0.87
	BT-as	55.7	92.9	5.6	4.5	40.4	0.88
	HF-bs	58.8	58.1	11.9	4.8	39.6	0.88
	HF-as	55.6	94.1	5.4	4.5	40.5	0.88
	LF-bs	58.0	67.1	10.2	4.7	39.8	0.88
	LF-as	54.7	104.3	3.6	4.4	40.7	0.88
	UL-bs	58.1	65.5	10.5	4.8	39.8	0.88
	UL-as	55.5	94.8	5.3	4.5	40.5	0.88
	WH-bs	57.0	78.3	8.2	4.7	40.1	0.88
	WH-as	55.8	92.1	5.7	4.5	40.4	0.88

Note: *Chlorella vulgaris* (CV) and *Chlorella protothecoides* (CP) were cultivated on five media: one synthetic (Bristol medium, BT) and four prepared using effluents from different anaerobic digesters treating food waste at a high (HF; 5 g VS/L·d) and a low (LF; 1.5 g VS/L·d) organic loading rates, *Ulva* (UL), and whey (WH), respectively.

[a] CN, cetane number; IV, iodine value; CP, cloud point; KV, kinematic viscosity; HHV, higher heating value; SG, specific gravity

[b] Not applicable.
